# Emergent nanoscale superparamagnetism at oxide interfaces

**DOI:** 10.1038/ncomms12566

**Published:** 2016-08-25

**Authors:** Y. Anahory, L. Embon, C. J. Li, S. Banerjee, A. Meltzer, H. R. Naren, A. Yakovenko, J. Cuppens, Y. Myasoedov, M. L. Rappaport, M. E. Huber, K. Michaeli, T. Venkatesan, Ariando, E. Zeldov

**Affiliations:** 1 Department of Condensed Matter Physics, Weizmann Institute of Science, Rehovot 7610001, Israel; 2 NUS Graduate School for Integrative Sciences and Engineering, National University of Singapore, Singapore 117456, Singapore; 3 NUSNNI-NanoCore, National University of Singapore, Singapore 117411, Singapore; 4 Departments of Physics and Electrical Engineering, University of Colorado Denver, Denver, Colorado 80217, USA; 5 Department of Physics, National University of Singapore, Singapore 117542, Singapore; 6 Department of ECE and MSE, National University of Singapore, Singapore 117576, Singapore

## Abstract

Atomically sharp oxide heterostructures exhibit a range of novel physical phenomena that are absent in the parent compounds. A prominent example is the appearance of highly conducting and superconducting states at the interface between LaAlO_3_ and SrTiO_3_. Here we report an emergent phenomenon at the LaMnO_3_/SrTiO_3_ interface where an antiferromagnetic Mott insulator abruptly transforms into a nanoscale inhomogeneous magnetic state. Upon increasing the thickness of LaMnO_3_, our scanning nanoSQUID-on-tip microscopy shows spontaneous formation of isolated magnetic nanoislands, which display thermally activated moment reversals in response to an in-plane magnetic field. The observed superparamagnetic state manifests the emergence of thermodynamic electronic phase separation in which metallic ferromagnetic islands nucleate in an insulating antiferromagnetic matrix. We derive a model that captures the sharp onset and the thickness dependence of the magnetization. Our model suggests that a nearby superparamagnetic–ferromagnetic transition can be gate tuned, holding potential for applications in magnetic storage and spintronics.

## Abstract (web summary)

Interfaces between complex oxides can exhibit diverse emergent phenomena, such as magnetic and superconducting order. Here, the authors evidence the emergence of nanoislands with a thickness dependent transition from superparamagnetic to ferromagnetic behaviour at LaMnO_3_/SrTiO_3_ thin film interfaces.

## Graphical abstract

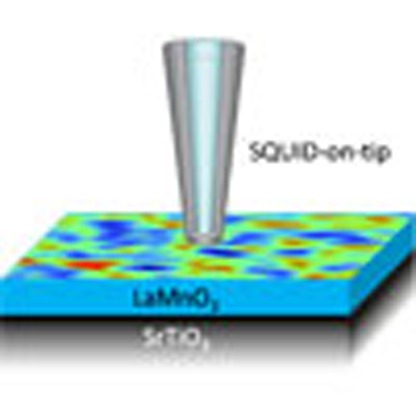

In recent years the extensive efforts to create new types of heterostructures based on transition metal oxides have come to fruition^1^,^2^,^3^,^4^,^5^,^6^,^7^,^8^. The strong interactions characterizing these materials are an appealing feature as they provide a nurturing platform for new phases^9^. Interestingly, in the most extensively studied LaAlO_3_/SrTiO_3_ (LAO/STO) system, the non-magnetic insulator parent compounds give rise to conductivity^1^,^7^, magnetism^10^,^11^,^12^,^13^,^14^,^15^ and superconductivity^2^,^14^,^15^,^16^,^17^, which are sharply tuned by the number of LAO layers in the heterostructure. Motivated by the complex physics arising at the interfaces of band insulators, we studied LaMnO_3_ (LMO)/STO heterostructures, in which one parent compound (LMO) exhibits a very diverse phase diagram already in the bulk^18^. In undoped bulk LMO, orbital order due to Jahn–Teller distortions of the MnO_6_ octahedra sets in at high temperatures of ∼750 K. Subsequently, magnetic exchange between Mn^3+^ ions leads to formation of A-type antiferromagnetic (AFM) phase with Néel temperature of ∼140 K in which ferromagnetic (FM) planes are aligned antiferromagnetically^19^,^20^. At high doping or at high oxygen content, bulk LMO undergoes a phase transition into a FM metallic state^19^,^21^,^22^,^23^. The nature of the magnetic phases in thin films is, however, more controversial. While stoichiometric LMO is AFM in the bulk, a number of studies describe the appearance of FM behaviour even in stoichiometric thin films^24^,^25^,^26^,^27^,^28^,^29^,^30^. In addition, while FM state in the bulk is metallic, an insulating FM behaviour was reported for thin films^25^,^26^,^27^. Hence, the interplay between charge, orbital and spin degrees of freedom combined with low dimensionality and the fact that LMO has a polar structure similar to LAO, offers exciting potential for new emergent phenomena in superlattices and heterostructures^20^,^27^,^31^,^32^. In particular, since the existence of magnetism and its possible origins in LAO/STO remain controversial^5^,^33^, the prospect of realization of tunable magnetic order in LMO/STO is of high fundamental and technological interest.

We report here the observation of an abrupt transition from an AFM phase to a highly inhomogeneous magnetic state when more than five unit cells (u.c.) of LMO are epitaxially grown on the STO. Using a scanning superconducting quantum interference device (SQUID) microscope we probed the nature of the magnetic state. The imaging measurements reveal well separated and weakly correlated superparamagnetic (SPM) islands. These nanoscale magnetic puddles account for the entire magnetization of the LMO/STO heterostructure as observed in global measurements. We interpret the experimental findings as electronic phase separation leading to the nucleation of metallic nanoscale ferromagnetic islands embedded in an insulating antiferromagnetic matrix. We propose a theoretical model based on electronic reconstruction due to the polar nature of the interface that captures the essential part of our experimental results.

## Results

### Magnetic structure in zero-field cooled state

**Figure 1:**
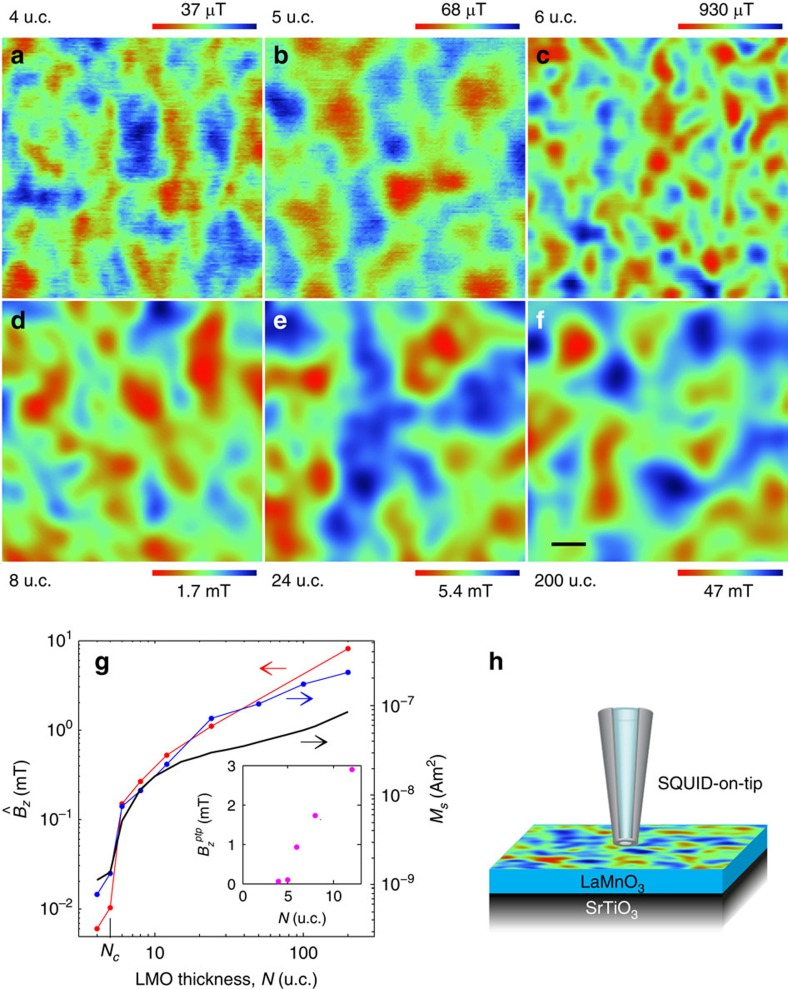
ZFC magnetic structure of LMO/STO of different thicknesses. (**a**–**f**) 1.5 × 1.5 μm^2^ scanning SOT images of the magnetic field *B*_*z*_(*x*,*y*) of six samples of different thicknesses *N*=4 to 200 u.c. at ∼100 nm above the surface acquired at 4.2 K. Scale bar is 200 nm. Note that in (**a**) through (**f**) the color scale changes by more than three orders of magnitude. (**g**) Thickness dependence of the r.m.s. value 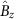 in scanning SOT images (red), global magnetization saturation value *M*_*s*_ (blue), and theoretically calculated *M*_*s*_ (black) which takes into account the SPM and the AFM canting contributions. The inset shows the peak-to-peak field variation *B*_*z*_^*ptp*^ in SOT images versus *N* near *N*_*c*_=5. (**h**) Schematic scanning SOT microscopy setup.

Here we use a scanning probe microscope based on a nanoscale SQUID that resides on the apex of a quartz tip (SQUID-on-tip (SOT))^34^,^35^ for imaging of the local magnetic structure in stoichiometric LMO films grown epitaxially on TiO_2_-terminated (001) STO substrates (see Methods section). Figure 1a–f show images of the out-of-plane magnetic field *B*_*z*_(*x*,*y*) in LMO films of various thicknesses acquired at a height of ∼100 nm above the sample surface at 4.2 K after zero-field cooling (ZFC). A highly inhomogeneous *B*_*z*_(*x*,*y*) is observed in all the films with a characteristic scale of 100–200 nm, comparable to the size of our SOTs (90–230 nm diameter, Supplementary Table 1). Remarkably, even though the *B*_*z*_(*x*,*y*) structures look similar, the span of the local field varies by more than three orders of magnitude between the samples (see colour bars). Figure 1g shows the quantitative analysis of the r.m.s., 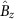, and peak-to-peak, 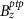 (inset), values of *B*_*z*_(*x*,*y*) versus the thickness *N* (in u.c.) of the LMO film. For *N*≤*N*_*c*_=5, 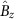 and 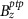 remain very small. However, at *N*=6 a discontinuous change in their values by more than an order of magnitude occurs. A similar behaviour, although on a much larger length scale, was recently reported by Wang *et al*.^27^ and interpreted as a phase transition from AFM to FM state driven by the polar electronic reconstruction. Interestingly, the saturation value *M*_*s*_ obtained from global magnetization measurements at elevated fields (Supplementary Fig. 1) shows similar *N* dependence (Fig. 1g, blue dots), further suggesting a sharp onset of FM order in LMO/STO. It is worth noting the same features were found everywhere across the sample (see Supplementary Note 1 and Supplementary Fig. 2).

### Magnetization reversal process

**Figure 2:**
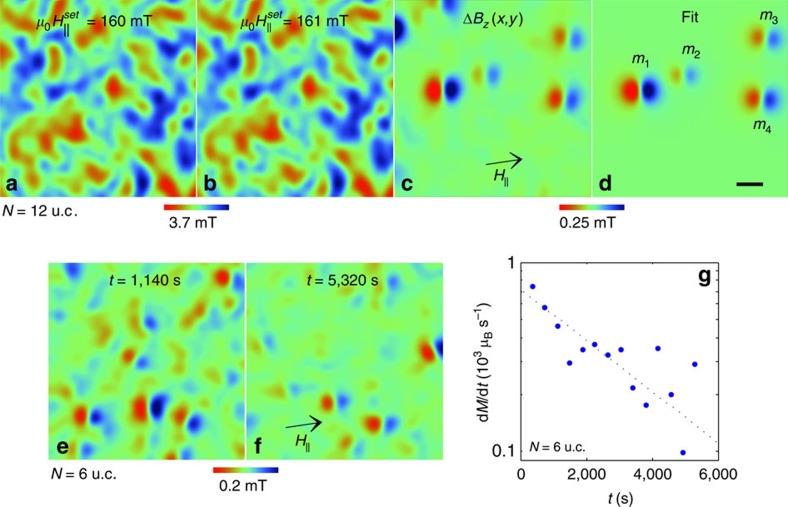
Field-driven and temporal magnetization reversal dynamics in LMO/STO. (**a**,**b**) Two consecutive 1.5 × 1.5 μm^2^ images of *B*_*z*_(*x*,*y*) (see Supplementary Movie 2) in an *N*=12 u.c. sample after a field excursion to 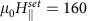 (**a**) and 161 mT (**b**). (**c**) Differential image Δ*B*_*z*_(*x*,*y*) obtained by direct subtraction of (**a**) from (**b**) revealing magnetization reversal events of isolated SPM nanoscale islands (see Supplementary Movie 3). Note an order of magnitude enhanced colour scale. (**d**) Numerical fit to Δ*B*_*z*_(*x*,*y*) in (**c**) with four in-plane oriented islands using SOT diameter of 104 nm, scan height of 105 nm, and *N*_*e*_=2 u.c. with resulting magnetic moments *m*_1_=1.0 × 10^5^ μ_B_ (*D*_1_=40 nm), *m*_2_=2.6 × 10^4^ μ_B_ (*D*_2_=20 nm), *m*_3_=4.7 × 10^4^ μ_B_ (*D*_3_=28 nm), and *m*_4_=6.8 × 10^4^ μ_B_ (*D*_4_=33 nm). Scale bar is 200 nm. (**e**) Δ*B*_*z*_(*x*,*y*) image in *N*=6 u.c. sample showing the thermally activated magnetization reversals of the islands at a constant 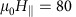 mT at *t*=1,140 s after the field ramp (see Supplementary Movie 6). (**f**) Same as (**e**) at *t*=5,320 s. (**g**) The magnetization relaxation rate d*M*/d*t* attained by vectorial summation of the reversal events *m* in each frame of Supplementary Movie 6. Dotted line is a guide to the eye.

**Figure 3:**
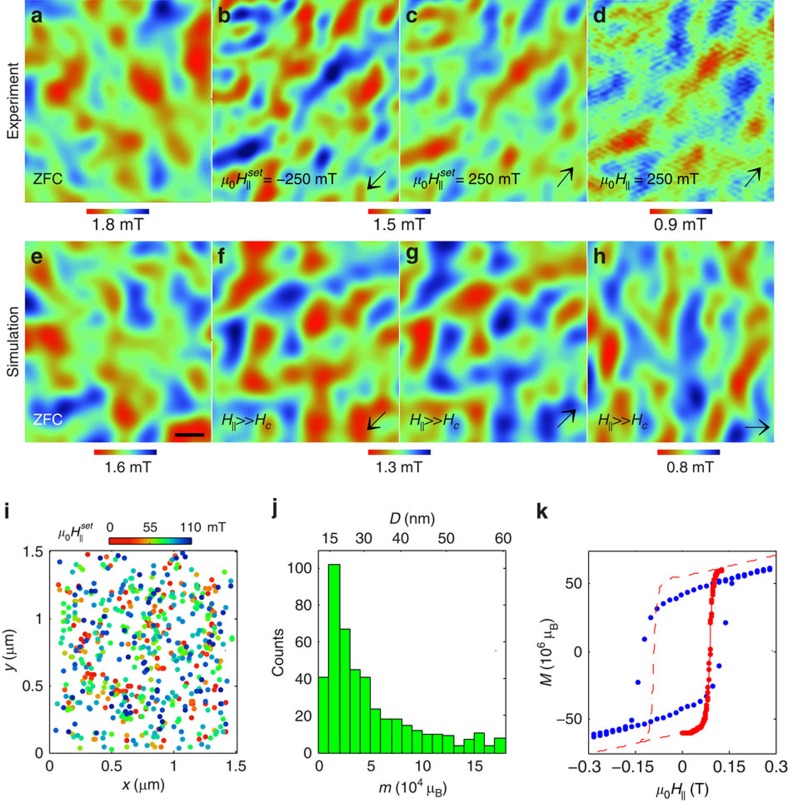
Saturated magnetic state and magnetization reversal process in *N*=8 unit cells sample. (**a**–**d**) *B*_*z*_(*x*,*y*) images of 1.3 × 1.4 μm^2^ after ZFC (**a**), after excursion to 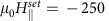 mT (**b**), after excursion to 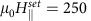 mT (**c**), and in the presence of 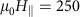 mT (enhancing instrumental noise) (**d**). The dark arrows show the 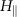 direction in each image. Note the strong anti-correlation between (**b**,**c**) indicating the full magnetization reversal of the SPM islands. The differences in the intensity in (**b**–**d**) are due to a drift in the scanning height of the SOT. (**e**–**h**) Numerical simulations of *B*_*z*_(*x*,*y*) 105 nm above the sample arising from randomly positioned non-overlapping SPM islands at the top and bottom surfaces of the 8 u.c. film using the measured *m* distribution in (**j**). (**e**) ZFC state with random magnetization orientation of SPM islands along *x* and *y* easy axes. Scale bar is 200 nm. (**f**–**h**) Same as (**e**) with all the moments oriented with equal probability either along −*x* or −*y* (**f**), along +*x* or +*y* (**g**), and only along +*x* (**h**) directions. (**i**) *x−y* locations of the SPM reversal events with the colour referring to the field 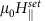 at which the reversal occurred (see Supplementary Movie 4). (**j**) Histogram of the magnetic moments *m* of the SPM reversals in (**i**) and the corresponding island diameters *D* (top axis) using *N*_*e*_=2 u.c. (**k**) Cumulative magnetic moment *M* versus applied field (red) attained by vectorial summation of all the reversal events *m* after sweeping the field from large negative value. The dashed line shows corresponding schematic reconstruction of *M*(*H*). Global *M*(*H*) measurement (blue, after subtraction of bare STO *M*(*H*)) normalized by the total sample area relative to the imaged area.

Since global measurements show that LMO/STO films have an in-plane magnetization^27^ (Supplementary Note 2), we have studied *B*_*z*_(*x*,*y*) while progressively increasing the in-plane magnetic field 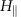 (Supplementary Movies 1 and 2). For each set value of 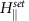, *B*_*z*_(*x*,*y*) was acquired either in the presence of 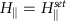 or after reducing 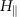 back to zero (to minimize noise and drift), with similar results. Figure 2a,b shows an example of two consecutive *B*_*z*_(*x*,*y*) images from Supplementary Movie 2 in *N*=12 u.c. sample for 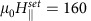 and 161 mT, which appear to be identical. To uncover the underlying magnetization process, we numerically subtract Fig. 2a from Fig. 2b, revealing small isolated dipole-shaped features in the differential image Δ*B*_*z*_(*x*,*y*) in Fig. 2c and in the corresponding Supplementary Movie 3. Figure 2d presents the numerical fit (see Methods section) of Δ*B*_*z*_(*x*,*y*) demonstrating that the dipole-shaped features are magnetization reversals of isolated nanoscale islands with in-plane magnetic moment *m*. As 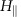 is increased, isolated islands undergo moment reversals at random locations, as presented in Fig. 3i and Supplementary Movie 4, indicating weak inter-island interactions. We did not observe magnetization reversal events for sample with *N*≤*N*_*c*_=5 u.c. (Supplementary Fig. 3).

### Superparamagnetic state

The observed magnetic behaviour is profoundly different from that expected for a FM state. A FM film after ZFC is composed of macroscopic domains with different magnetization orientations separated by domain walls (DW). As the field is increased, a magnetization process occurs through motion of DW: domains aligned along the magnetic field expand while oppositely aligned ones shrink, until a homogeneous state is achieved. Thus, the process of local microscopic magnetization reversal is highly correlated in space as DWs propagate through the sample. In the LMO/STO heterostructures, in contrast, there is no DW motion as seen in Supplementary Movies 1 and 2. Instead, the sample behaves as if it was composed of a sparse array of weakly interacting single-domain FM nanoparticles or islands. Since each nanoparticle has a different coercive field, the magnetization process occurs through uncorrelated reversal events of the individual islands at random locations (Supplementary Movie 4). This behaviour is analogous to slow magnetization dynamics in a two-dimensional array of SPM nanoparticles at temperatures below their blocking temperature *T*_*B*_, leading to a hysteretic magnetization loop^36^. Moreover, if the SPM islands are densely packed with a complete areal coverage, the resulting field distribution in the fully magnetized state should be uniform like in a fully polarized FM film. In contrast, Fig. 3a–d shows that the LMO films display highly non-uniform *B*_*z*_(*x*,*y*), that is similar to the ZFC *B*_*z*_(*x*,*y*), even in the fully magnetized state at fields much larger than the coercive field *H*_*c*_. This finding shows that the SPM islands are well separated from each other with substantial gaps between the islands that are nonmagnetic or only weakly magnetic. This physical separation renders the magnetic islands to form an essentially non-interacting SPM system rather than an interacting spin glass state^37^.

### Analysis of the SPM islands

The statistical analysis of *N*=8 u.c. sample in Fig. 3j shows that the magnetic islands have a typical moment of *m*≅1.5 × 10^4^ μ_B_, comparable to magnetic moments of common SPM nanoparticles^36^. By summing all the moments *m* of the flipped islands as a function of magnetic field, we find that the total in-plane moment 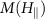 and its saturation value *M*_*s*_ measured microscopically over a very small area (∼8 × 10^−8^ of the 5 × 5 mm^2^ sample area) well account for the global magnetization of the sample as shown in Fig. 3k. The reversal process of the individual nanoscale islands thus provides a quantitative description of the macroscopic magnetization behaviour of LMO/STO heterostructures.

Taking the experimental histogram of Fig. 3j, we simulate the ZFC and the fully magnetized state by random spatial distribution of non-overlapping islands with in-plane moments *m* oriented either randomly (Fig. 3e) or fully aligned (Fig. 3f–h) (see Methods section). The resulting *B*_*z*_(*x*,*y*) in Fig. 3e–h closely describes the amplitude and the characteristic length scale of the ZFC state in Fig. 3a as well as of the non-uniform *B*_*z*_(*x*,*y*) in the magnetized state in Fig. 3b–d, emphasizing the presence of well-separated magnetic islands.

Reversal events were observed predominantly along the horizontal and vertical axes (see Supplementary Note 3 and Supplementary Fig. 4). The in-plane magnetization thus shows fourfold anisotropy with easy axes along the LMO crystallographic direction that are locked to the underlying STO crystal structure.

### SPM dynamics

Our local measurements are carried out at *T*=4.2 K well below the blocking temperature *T*_*B*_ which can be estimated from the temperature dependence of the global magnetization (Supplementary Fig. 5). Therefore, the fast temporal SPM dynamics are expected to be suppressed, leading to hysteresis in d.c. magnetization measurements^36^ (Fig. 3k and Supplementary Fig. 1). To investigate the existence of slow dynamics, 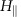 was abruptly raised and a sequence of *B*_*z*_(*x*,*y*) images was acquired at 380 s intervals in *N*=6 u.c. sample at constant 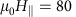 mT (Supplementary Movie 5). The corresponding Δ*B*_*z*_(*x*,*y*) images (Fig. 2e,f and Supplementary Movie 6) reveal the presence of a random moment reversal process following the fast field increase and leading to a slow relaxation of the overall magnetization. The rate of the moment relaxation d*M*/d*t* decays monotonically with time (Fig. 2g) as anticipated for tunnelling or thermal activation in a tilted potential. As in the case of the *B*_*z*_(*x*,*y*) dependence on 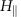, the observation of the temporal process of uncorrelated nanoscale magnetization reversals is in sharp contrast with DW motion in FM films. Here the nanoscale relaxation process is a direct manifestation of the slow dynamics of SPM islands at *T*<*T*_*B*_.

### Evaluation of the size of the SPM islands

Two possible doping mechanisms that may account for the magnetism in LMO/STO heterostructures will be discussed below: extrinsic bulk doping and surface charge reconstruction. We can estimate the characteristic size of the islands in these two cases assuming a magnetization of 4 μ_B_ per Mn atom and u.c. of 0.39 nm. In bulk LMO a spherical island with characteristic *m*=1.5 × 10^4^ μ_B_ (Fig. 3j) should thus have a diameter of *D*=7.5 nm, which is larger than the thickness *d*=3.1 nm of the 8 u.c. sample. In thin films with extrinsic bulk doping we therefore expect the islands to have a disk-like shape extending from top to bottom surfaces resulting in characteristic island diameter of *D*=9.5 nm in the 8 u.c. film. In the charge reconstruction model, in contrast, the magnetism occurs only within a surface layer of thickness *N*_*e*_. Taking *N*_*e*_=2 u.c. for the 8 u.c. sample (see derivation below), results in characteristic diameter of *D*=19 nm. The top axis in Fig. 3j shows the distribution of the island diameters in this case. Since both bulk and surface mechanisms lead to island sizes that are smaller than our spatial resolution of ∼100 nm determined by the SOT diameter and the scanning height, our local magnetic imaging cannot directly discern the two models.

### Theoretical models

We now elaborate on the possible mechanisms that can lead to the formation of the observed SPM state. The Mn^3+^ ions, which are responsible for the LMO magnetism, have four 3*d* valence electrons in three low-energy *t*_2*g*_ and one *e*_*g*_ orbital^19^. The spin-aligned *t*_2*g*_ electrons form a core spin of *S*=3/2, and the fourth electron, occupying the split *e*_*g*_ orbitals, is slaved to the core spins by Hund's coupling. The ground state is an A-type AFM Mott insulator with alternating planes of opposite magnetization of 4 μ_B_ per Mn atom^19^ (Supplementary Fig. 6). Hund's coupling between the Mn core spins and *e*_*g*_ electrons impedes hopping of any excess charges between AFM aligned sites, while FM alignment of the core spins allows excess charges to delocalize, thus lowering their kinetic energy^19^. Upon electron or hole doping, this double-exchange mechanism should therefore lead to a first-order phase transition from insulating AFM to metallic FM. At low doping, however, it has been shown that the energy of the system can be lowered by an electronic phase separation in which nanoscale FM islands are nucleated in the AFM matrix^38^. In particular, as derived in Supplementary Note 4 and shown in Supplementary Fig. 7, the phase separated state in LMO should be present for doping levels 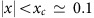 e per u.c., giving rise to metallic FM islands of 4–20 nm diameter containing an excess charge density 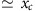. The observed SPM state could thus be a direct manifestation of the electronic phase separation due to low level of bulk carrier doping.

### Bulk doping mechanism

**Figure 4:**
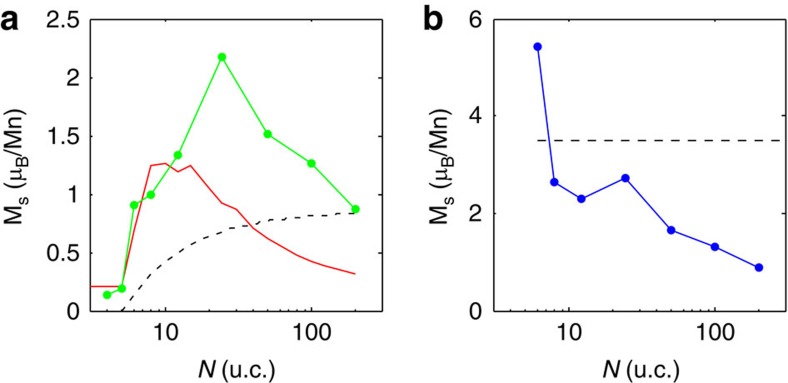
Magnetization per Mn atom versus film thickness. (**a**) Saturation magnetization derived from global measurements normalized by the number of Mn atoms in the entire thickness *N* of the films (green), the theoretically calculated average magnetization per Mn atom taking into account the SPM and the AFM canting contributions (red), and the average magnetization assuming a dead layer of *N*_*c*_=5 u.c. and 0.85 μ_B_ per Mn in the rest of *N*−*N*_*c*_ live layers (dotted). (**b**) Saturation magnetization derived from global measurements like in (**a**) but normalized by the number of Mn atoms in the *N*−*N*_*c*_ live layers assuming zero magnetization in the *N*_*c*_ dead layers (blue). The dotted line shows magnetization of 3.5 μ_B_ per Mn in the live layers as expected in highly doped LMO.

In LMO films, excess carriers in the bulk of the film can be introduced extrinsically either by chemical doping, like in the case of Sr-doped LMO where magnetization reaching 3.5 μ_B_ per Mn (close to the theoretical value) was measured in La_0.7_Sr_0.3_MnO_3_ films^39^, or by excess oxygen (cation deficiency) incorporated during the growth process^22^,^25^,^26^,^28^. Since all the samples were grown under the same conditions, one would expect the magnetization per Mn atom due to extrinsic bulk doping to remain independent of the LMO thickness *N*. Figure 4a shows the saturation magnetization per Mn atom versus *N* (green). For *N*=4 and 5 u.c. the magnetization is very small, but then increases sharply at *N*=6 to 0.9 μ_B_ per Mn, reaching a maximum of 2.2 μ_B_ per Mn at *N*=24, followed by a drop to 0.85 μ_B_ per Mn at *N*=200. Such a highly nonmonotonic behaviour combined with the vanishing magnetization for *N*≤*N*_*c*_ essentially rules out uniform doping.

Alternatively, in highly doped La_0.7_Sr_0.3_MnO_3_ films there is experimental evidence^39^ for spatial separation into two stacked sheets: a dead layer of few u.c. at the bottom with no magnetization and uniformly magnetized live layers on top. Taking into account a dead layer of *N*_*c*_=5, Fig. 4b shows the measured magnetization per Mn in the remaining *N*−*N*_*c*_ live layers. In contrast to the expected constant magnetization (dotted), the experimental data (blue) show a sharp onset of magnetization at *N*=*N*_*c*_+1 with 5.4 μ_B_ per Mn that decays to 0.85 μ_B_ per Mn for *N*=200 u.c., which renders this possibility unlikely. In addition, the magnetization of our thickest sample sets an upper bound for the extrinsic bulk saturation magnetization in our samples. Then, one would expect the average magnetization of thinner films in presence of a dead layer to be always below this value and grow asymptotically as demonstrated by the dotted line in Fig. 4a. In contrast, among all films with *N*>*N*_*c*_ the smallest magnetization is found in the thickest sample (200 u.c.). Moreover, compared with previous systematic studies^25^,^26^, a bulk magnetization of 0.85 μ_B_ per Mn indicates that our films are stoichiometric with very low extrinsic doping consistent with our Rutherford Backscattering Spectrometry and Particle Induced X-ray Emission PIXE measurements (Li and Ariando—private communication).

### Charge reconstruction model

Even though more complicated thickness-dependent effects including strain, growth defects and surface segregation could possibly give rise to nontrivial thickness dependence of the magnetization due to extrinsic doping, we propose here a simple alternative model. In the closely related LAO/STO heterostructures, electronic reconstruction driven by the polar catastrophe is believed to be responsible for a sharp onset of interface conductivity^4^. Since the LMO on TiO_2_-terminated STO has the same polar structure as of LAO/STO, a similar electronic reconstruction should be expected to occur also in LMO/STO heterostructures. Based on this analogy, Wang *et al*.^27^ recently proposed that a FM state forms at the LMO/STO interface above a critical thickness *N*_*c*_. We expand here this approach and show that electronic reconstruction should in fact lead to a SPM rather than FM state.

**Figure 5:**
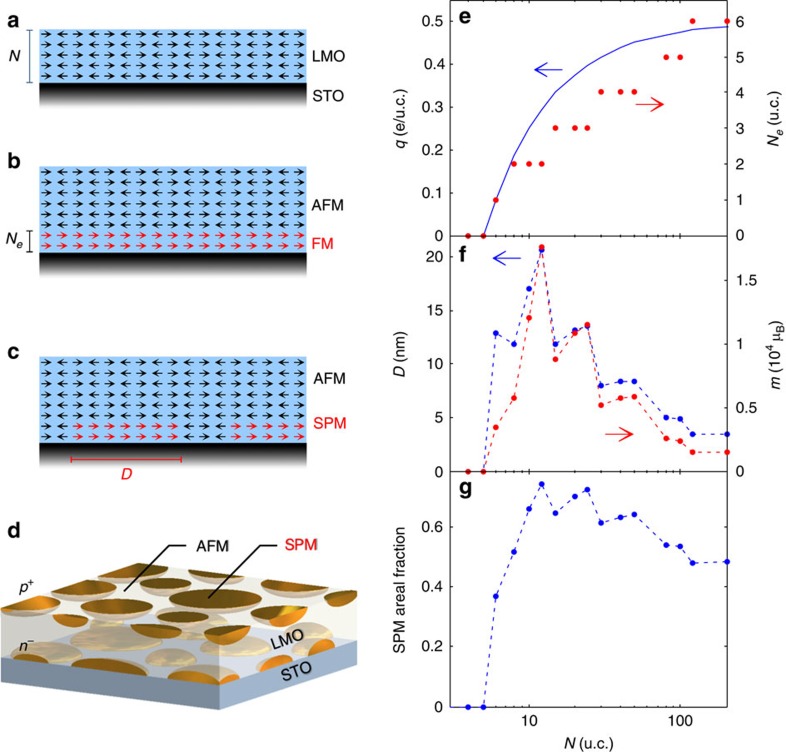
Theoretical model results and schematics. (**a**) Schematics of core Mn^3+^ spins in A-type AFM configuration (black arrows) in LMO at *N*≤*N*_*c*_=5. (**b**) For high electron concentrations at the LMO/STO interface, a metallic layer of thickness *N*_*e*_ with uniform FM order (red arrows) should be formed at the interface. (**c**) Theoretically derived phase separated state for *N*>*N*_*c*_ with SPM islands embedded in AFM matrix. (**d**) Schematic representation of hole-doped and electron-doped SPM islands in the case of electron transfer from Mn^3+^ orbitals at the top surface of LMO. (**e**) Calculated excess charge transfer per unit area *q*(*N*)=0.5(1−*N*_*c*_/*N*) (blue) and the thickness of electron charge layer *N*_*e*_ (red) versus LMO thickness *N*. (**f**) The diameter (blue) and the magnetic moment (red) of the SPM islands versus *N* in the phase separated state. (**g**) The areal fraction of the SPM islands versus *N*.

Since the bandgap of LMO is smaller than that of STO, charge reconstruction leads to electron transfer from the top surface to the Mn orbitals at the LMO side of the LMO/STO interface^27^. The number of transferred electrons grows with *N*>*N*_*c*_ until it asymptotically reaches areal density of 0.5 excess electrons per u.c. (Fig. 5e). The double-exchange mechanism in the presence of excess electrons at the interface is expected to induce a transition from an insulating AFM (Fig. 5a) to a metallic FM state (Fig. 5b). Our calculation reveals, however, that a new ground state with spontaneous phase separation emerges (Supplementary Note 4 and Supplementary Fig. 8). In this state segregated nanoscale FM metallic islands of excess electrons with *ρ*≅0.17 e per u.c. are formed at the interface within the undoped insulating AFM matrix (Fig. 5c). The competition between kinetic energy, Coulomb interactions and the core spin exchange interaction determines the separation and size of the islands. Self-consistent calculation of the electrostatic potential shows that the metallic FM islands are confined to the vicinity of the interface, spreading over a finite thickness *N*_*e*_ of a few u.c. (Fig. 5e and Supplementary Fig. 9). For *N*=12, the islands reach *D*=20 nm and *m*=1.6 × 10^4^ μ_B_ (Fig. 5f), which is in good agreement with the typical size and magnetic moment found in the experiment (Fig. 3j). Importantly, our model predicts that the phase separation occurs for all thicknesses (*N*>*N*_*c*_) of LMO and the areal fraction of the magnetic islands remains below 1 (Fig. 5g). Since the islands are of nanometer size and well separated from each other, the system always remains in an insulating SPM state. Intriguingly, if the excess electrons at the LMO/STO interface originate from Mn orbitals at the LMO surface rather than from oxygen vacancies^40^,^41^, a double-layer of hole-doped and electron-doped islands will form as depicted in Fig. 5d.

In thin samples (*N*≤*N*_*c*_) the weak magnetization arises from canting in the AFM phase^19^,^42^. Correspondingly, the low *B*_*z*_(*x*,*y*) shown in Fig. 1a,b apparently originates from AFM domains with different canting orientations^42^. The electronic reconstruction at *N*>5 induces a transition into the SPM phase at the interfaces which results in a sharp onset of magnetization. Moreover, the regions between the SPM islands as well as the central part of the films remain AFM and should, therefore, contribute about 0.2 μ_B_ per u.c. due to canting in the AFM phase^19^,^42^. This picture is further supported by the temperature dependence of *H*/*M* (Supplementary Fig. 10). The magnetic susceptibility obeys Curie–Weiss law with extrapolated Θ that is lower than the actual transition temperature, which apparently arises from bulk AFM response^23^ modified by the presence of the SPM islands. The saturation magnetization in Fig. 4a (red) was calculated taking into account the double-layer SPM islands and the bulk AFM contribution of 0.2 μ_B_ per u.c. Since charge transfer to the interface increases monotonously with *N* up to 0.5 e per u.c. (Fig. 5e), the overall saturation magnetization increases as well, as shown in Fig. 1g (black). However, the relative contribution of the interface SPM to the average magnetization per Mn atom peaks at intermediate thicknesses in good agreement with the experimental data (Fig. 4a).

## Discussion

The contribution of the electronic reconstruction mechanism is expected to diminish in the limit of thick samples. Consequently, the deviation between the calculated and the measured magnetization in the thicker samples in Fig. 4a indicates the presence of additional bulk contribution to the magnetization. Indeed, in the general case of low doping the magnetism due to electronic reconstruction should coexist with bulk magnetism, leading to SPM islands residing both in the bulk of the LMO films and on the surfaces. Interestingly, although microscopic phase separation in bulk manganites has been discussed in the literature^18^,^19^,^38^,^43^,^44^, its consequence of forming a SPM state has not been considered. Hence the finding of SPM has broad implications on our comprehension of magnetic and transport properties of LMO. In particular, our results imply that a macroscopic FM state should be attainable only at high doping, while SPM should prevail over a wide range of intermediate doping levels. Since the blocking temperature *T*_*B*_ of the SPM islands is rather high, the magnetization is hysteretic over a wide range of temperatures below *T*_*B*_, making the distinction between FM and SPM difficult based on global measurements. Furthermore, since a FM state is expected to be metallic, the SPM state in which nanoscale FM islands reside in an AFM matrix may provide the explanation for the existence of a FM insulating state in LMO films.

To conclude, the intriguing interplay of AFM, FM, and SPM and the controllable nanoscale phase separation provide the basis for novel physics and new functionalities in LMO/STO heterostructures. Our calculations show that a relatively small increase in carrier concentration can transform the insulating SPM into a conducting FM state opening the route to electric gate control of magnetism and magnetoresistance, with potential applications in magnetic recording and spintronics. LMO is thus an important addition to the oxide family of heterostructure materials offering novel possibilities for engineering of multifunctional multi-layers.

## Methods

### SOT imaging

The magnetic imaging was performed using Pb SOT devices^34^, a typical effective diameter of ∼100 nm and white noise of ∼200 nΦ_0_ Hz^−1/2^ (see Supplementary Note 5, Supplementary Fig. 11 and Supplementary Table 1). The SOT was mounted in a home-built scanning probe microscope using a series SQUID array amplifier^45^ for signal readout. Scanning was performed using Attocube integrated *xyz* scanner with *xy* range of 30 μm and *z* range of 15 μm. To bias the SOT to a sensitive point, an out-of-plane field *H*_⊥_ was applied (see Supplementary Table 1). No influence of *H*_⊥_ on the sample response was observed. All the measurements were performed at 4.2 K in He exchange gas at ∼1 mbar. The pixel size of all the SOT images shown in the main text is 5 × 5 nm^2^ and the acquisition time was about 5 min per image.

### Sample growth

The LMO films on TiO_2_-terminated single crystal STO (001) substrates were deposited by pulsed laser deposition from a polycrystalline LMO target in an oxygen partial pressure of 10^2^ mbar at 750 °C by using 1.8 J cm^−2^ pulses at 248 nm with a repetition rate of 2 Hz. The STO substrates (CrysTec GmbH, Berlin) of 5 × 5 mm^2^ and 0.5 mm thickness were double side polished and chemically treated in buffered hydrofluoric acid and annealed at 950 °C in oxygen, resulting in singly terminated STO surface with atomically flat terraces of single STO unit-cell height and terrace width of ∼300 nm. The layer-by-layer growth of the films was monitored in situ using reflection high-energy electron diffraction and the samples were cooled to room temperature in oxygen at the deposition pressure. See Supplementary Fig. 12 for representative atomic force microscopy images of *N*=12 and 24 u.c. LMO films showing atomic-step terraces are preserved, demonstrating the layer-by-layer growth of the LMO thin films.

### Global characterization

Global magnetization measurements of the samples were done using a Quantum Design magnetic properties measurement system vibrating sample magnetometer with temperature range of 2–300 K. The M–H loops of LMO films were obtained after subtraction of the STO diamagnetic background signal.

### Simulations of *B*_*z*_ field distribution

To simulate the local field distribution *B*_*z*_(*x*,*y*) in the SPM state, we use a random distribution of non-overlapping magnetic islands using the experimentally attained distribution of sizes and moments *m* in the 8 u.c. sample shown in Fig. 3j. Figure 3e shows the ZFC state in which the in-plane moment orientation was taken to be random along the *x* and *y* easy axes, as discussed in Supplementary Note 3. The shown *B*_*z*_(*x*,*y*) was calculated using the experimental values of SOT diameter of 114 nm and scan height of 105 nm. The result of Fig. 3e compares well with the experimental data in Fig. 3a both in the size of characteristic features and in the span of *B*_*z*_(*x*,*y*). Figure 3f shows *B*_*z*_(*x*,*y*) of the same sample as in Fig. 3e but with all the moments oriented randomly either in the −*x* or −*y* directions to simulate the fully magnetized case at *H*>*H*_*c*_ applied at 225° with respect to the +*x* axis. In contrast to the FM case, in which a uniform field is attained at full magnetization, the resulting *B*_*z*_(*x*,*y*) remains highly inhomogeneous because the SPM islands are well separated. Figure 3f shows that a fully magnetized SPM state displays *B*_*z*_(*x*,*y*) that is similar to the ZFC state with a moderate reduction in the field span consistent with the experimental data in Fig. 3a–d. An SPM state fully magnetized in the +*x* and +*y* directions (Fig. 3g) results in *B*_*z*_(*x*,*y*) that is identical to Fig. 3f but of opposite polarity, as observed experimentally in Fig. 3b,c. Similar result is attained by polarizing all the islands in the +*x* direction (Fig. 3h) albeit with slightly reduced field span since all the SPM island are oriented in a unique direction (+*x*) which is not case for Fig. 3f,g.

### Data analysis

For each sample, a sequence of *B*_*z*_(*x*,*y*) images was acquired, increasing 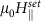 in steps of 1 mT from 0 to 250 mT after sweeping the field from−250 mT. After numerically detecting and correcting the relative drifts in the *xy* plane, each pair of images was subtracted to obtain Δ*B*_*z*_(*x*,*y*) (Fig. 2c). Each dipole-like feature in Δ*B*_*z*_(*x*,*y*) is assumed to originate from a SPM island that reversed its in-plane magnetic moment oriented at an angle *θ* from−*m* to +*m*. The value of *m* is determined by the diameter *D* and thickness *N*_*e*_ (taken from theory in Fig. 5e) of the island using magnetization of 4 μ_B_ per Mn with lattice parameter of 0.39 nm. Fitting is then performed with *D*, *θ* and *h* as free parameters including a convolution with the SOT size (keeping the same *N*_*e*_ and SOT height *h* for all islands). Figure 2d shows an example of the fit result. Since *D* is smaller than *h* and the SOT diameter, the resulting Δ*B*_*z*_(*x*,*y*) is essentially described by a point-like in-plane magnetic moment *m* almost independent of its size *D*. Therefore, the choice of *N*_*e*_ (taken from theory) affects the derived *D* but has little effect on the derived value of *m*.

### Data availability

All relevant data are available on request, which should be addressed to Y.A., Ariando or E.Z.

## Additional information

**How to cite this article:** Anahory, Y. *et al*. Emergent nanoscale superparamagnetism at oxide interfaces. *Nat. Commun.* 7:12566 doi: 10.1038/ncomms12566 (2016).

## Supplementary Material

Supplementary Information Supplementary Figures 1-12, Supplementary Table 1, Supplementary Notes 1-5 and Supplementary References

Supplementary Movie 1 Supplementary Movie 1 shows a sequence of 1.5 × 1.5 μm^2^ B_z_(*x*,*y*) images in the *N*=8 u.c. sample acquired upon increasing *μ*_0_*H*_‖_^set^ from 0 to 150 mT in steps of 1 mT applied at *θ*=52° relative to the *x* axis (and the [100] STO direction) after full magnetization of the sample at -250 mT. The first frames of the movie mainly show the instrumental *x*,*y* drift arising from application of *H*_‖_. With increasing *μ*_0_*H*_‖_^set^ subtle changes in B_z_(*x*,*y*) at random locations begin to be visible. These changes grow significantly on approaching *H_c_* ≈ 95 mT followed by reduction in the changes at higher fields. Note that the B_z_(*x*,*y*) images at the beginning and at the end of the movie are practically inverted, indicating that all the SPM islands have fully reversed their moments.

Supplementary Movie 2 Supplementary Movie 2 shows a similar process in the *N*=12 u.c. sample upon increasing *μ*_0_*H*_‖_^set^ from 0 to 250 mT at *θ*=7°. After the initial drift, random small changes become visible. Our maximal in-plane field of 250 mT is, however, insufficient to reach a full inversion of all the SPM islands.

Supplementary Movie 3 The corresponding Δ*B*_z_(*x*,*y*) images in the *N*=12 u.c. sample are shown in Supplementary Movie 3 for *μ*_0_*H*_‖_^set^ from 125 to 250 mT obtained by subtraction of consecutive images in Supplementary Movie 2 (note an order of magnitude smaller colour bar span). Randomly-appearing dipole-like features show the magnetization reversal process of SPM islands. Most of the dipole-like features are oriented close to the x direction along the easy magnetization axis of [100] STO.

Supplementary Movie 4 Supplementary Movie 4 shows the *x*,*y* coordinates of the SPM island reversal events (after drift correction) in the *N* = 8 u.c. sample as *H*_‖_^set^ is increased. The events show random uncorrelated behaviour consistent with the SPM state. The compilation of all the locations is presented in Fig. 3i.

Supplementary Movie 5 The thermally activated process of the magnetic reversal of the SPM islands is presented in Supplementary Movies 5 and 6 of *N* = 6 u.c. sample. The in-plane field was rapidly ramped to 80 mT and a sequence of *B*_z_(*x*,*y*) images (Supplementary Movie 5) was acquired at a constant *μ*_0_*H*_‖_ = 80 mT for about 1.5 h. The acquisition time of each image was 360 sec with 20 sec interval between the images.

Supplementary Movie 6 The sequence of Δ*B*_z_(*x*,*y*) images (Supplementary Movie 6) is attained by subtraction of consecutive *B*_z_(*x*,*y*) images. The dipole-like features in Supplementary Movie 6 show that following the rapid increase in *μ*_0_*H*_‖_ the islands continue to reverse their moments predominantly in the direction of *μ*_0_*H*_‖_ for an extended period of time through a thermally activated process. The number of the reversal events decays with time as seen in Supplementary Movie 6. The relaxation rate of the total in-plane magnetic moment d*M*/d*t* in the scanned area presented in Fig. 2g is obtained by vectorial summation of the reversing moments *m* along *H*_‖_ direction for each frame of Supplementary Movie 6.
