# Supplementary material for: Emergent nanoscale superparamagnetism at oxide interfaces
Source: Nat Commun. 2016 Aug 25;7:12566. doi: 10.1038/ncomms12566 (PMC5007328; doi:10.1038/ncomms12566)
Supplement: Supplementary Information — Supplementary Figures 1-12, Supplementary Table 1, Supplementary Notes 1-5 and Supplementary References [file ncomms12566-s1.pdf]

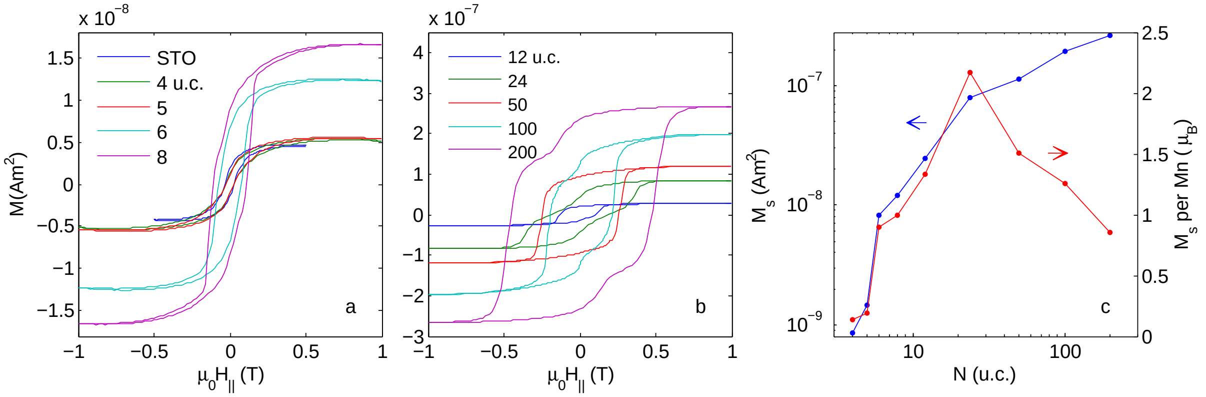

Supplementary Figure 1: **Global magnetic measurement and saturation magnetization.** (a,b) The in-plane magnetic moment $M(H)$ of 5×5 mm^2 LMO/STO samples of various indicated thickness vs. the applied in-plane field. (c) Saturation magnetic moment of the samples M_s (after subtraction of bare STO) (blue) and M_s per Mn atom (red) vs. thickness N.

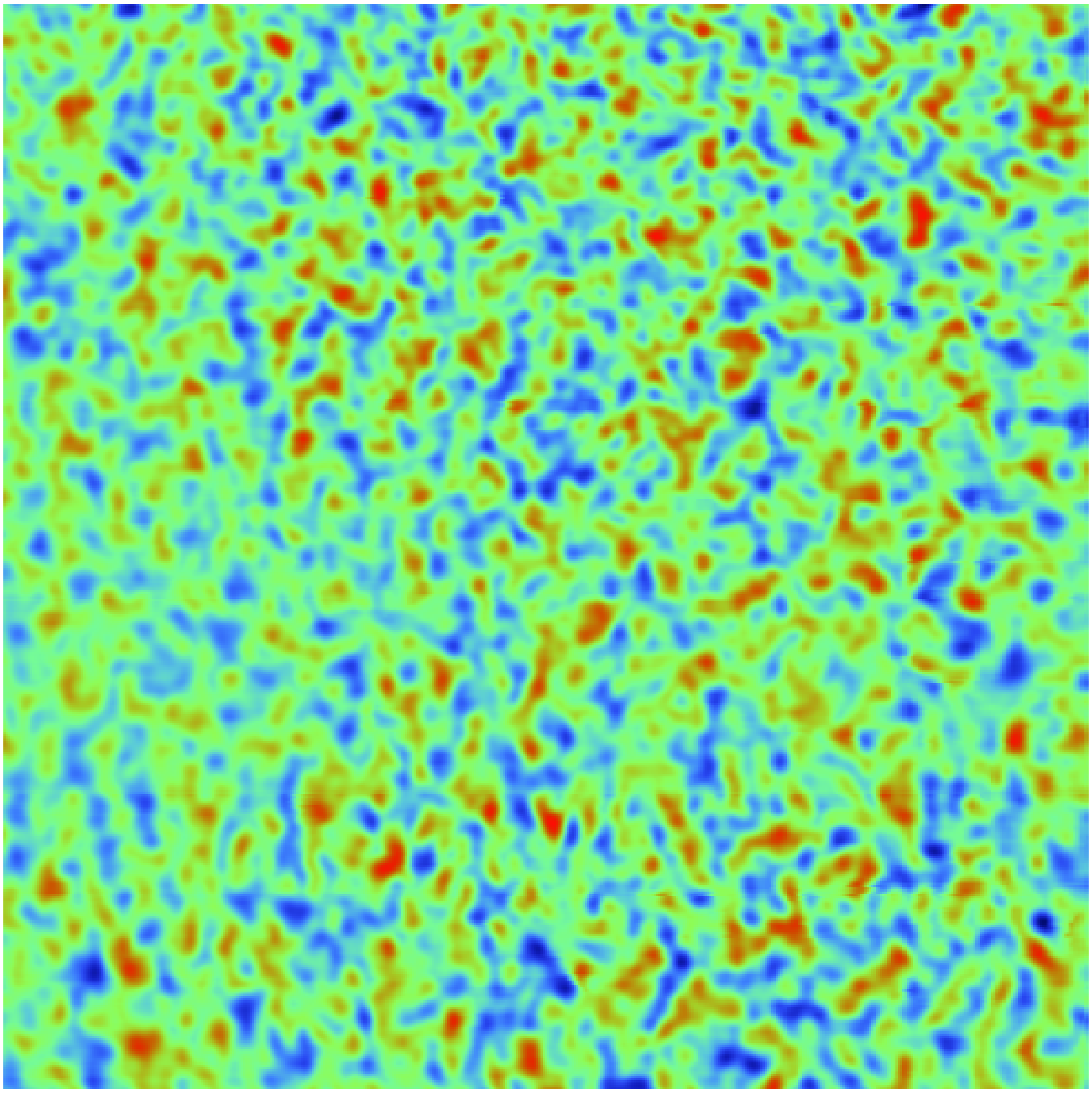

Supplementary Figure 2: **Larger scale image.** Large area $B_z(x, y)$ image of $10 \times 10\ \mu\text{m}^2$ after ZFC of $N = 12$ u.c. sample. The color scale spans 2.8 mT.

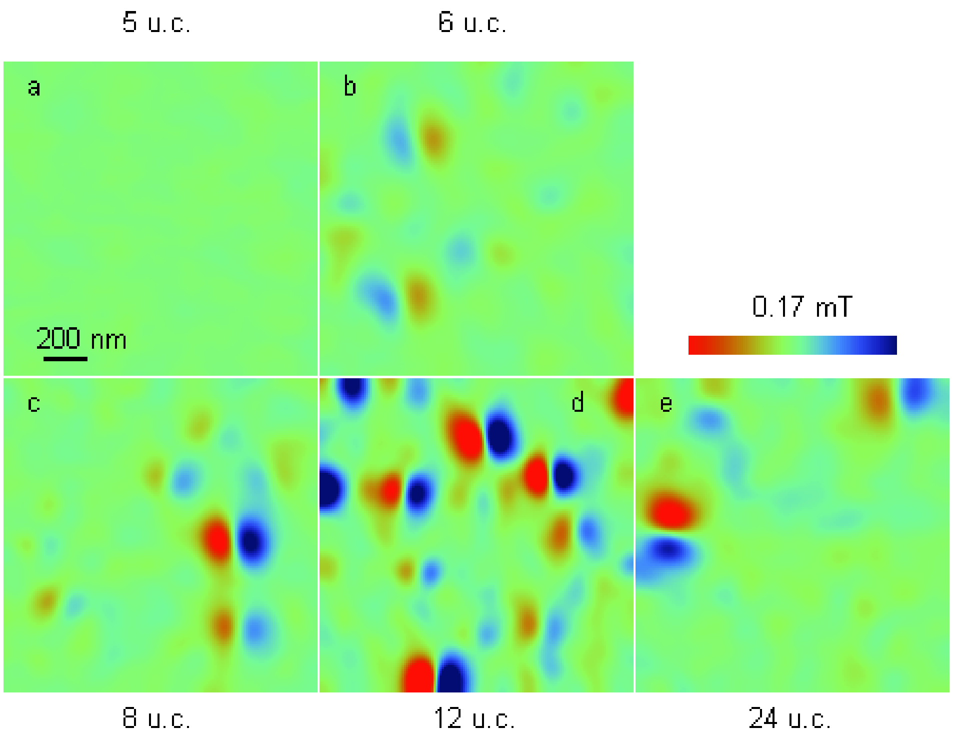

Supplementary Figure 3: Thickness dependence of $\Delta B_z(x, y)$. Representative $\Delta B_z(x, y)$ images in various LMO/STO samples. Dipole-like SPM reversal features are observed in all samples with $N > N_c = 5$ u.c. The images were attained by subtracting consecutive $B_z(x, y)$ images with 1 mT applied field intervals.

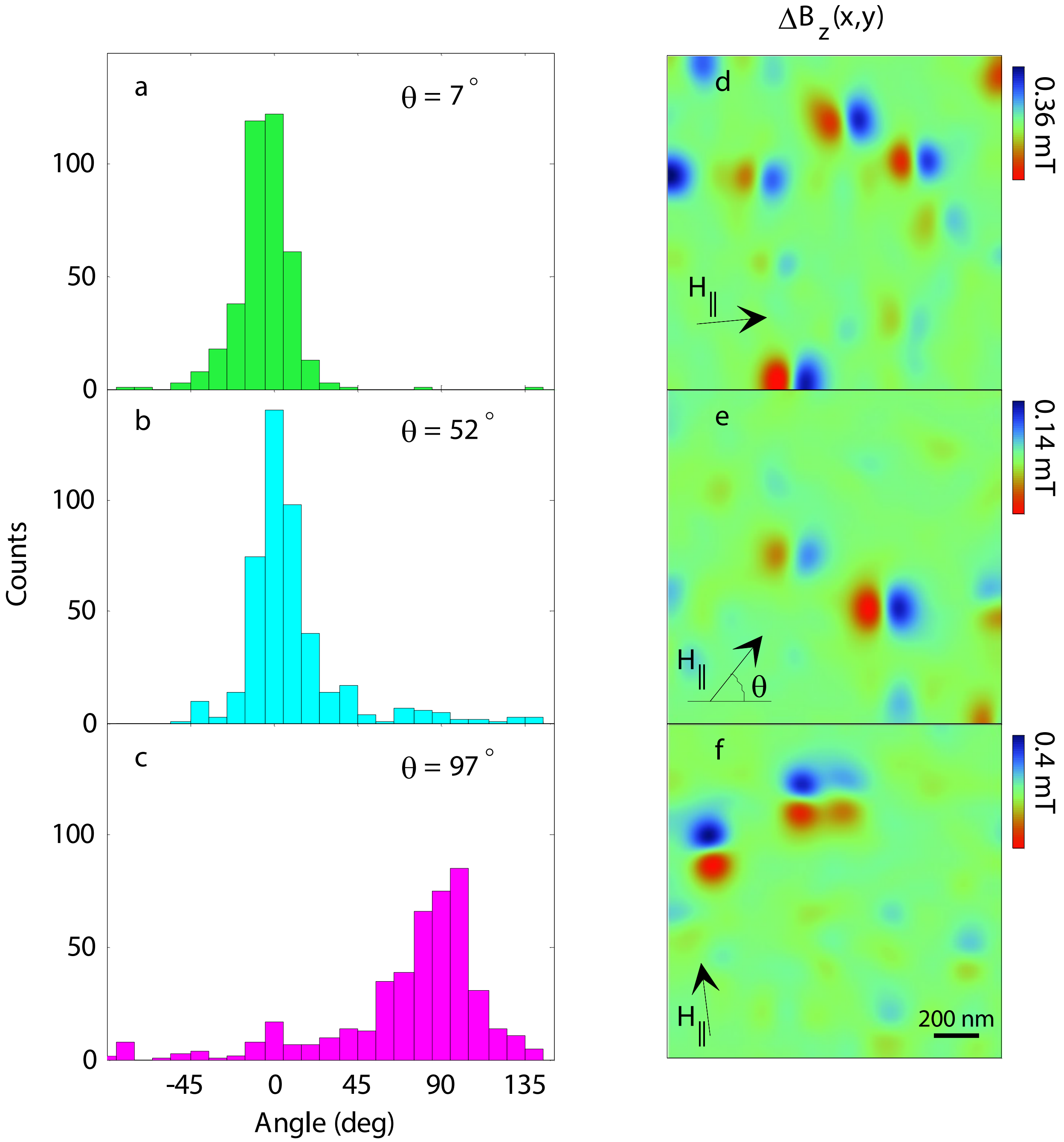

Supplementary Figure 4: **In-plane anisotropy.** (a-c) Histograms of the angular distribution of the SPM moment m orientations in $N = 12$ u.c. sample for three orientations of the applied field $\theta = 7^\circ$ (a), 52° (b), and 97° (c) relative to the [100] STO orientation. (d-f) Examples of corresponding $\Delta B_z(x, y)$ images showing various orientations of the moment reversals.

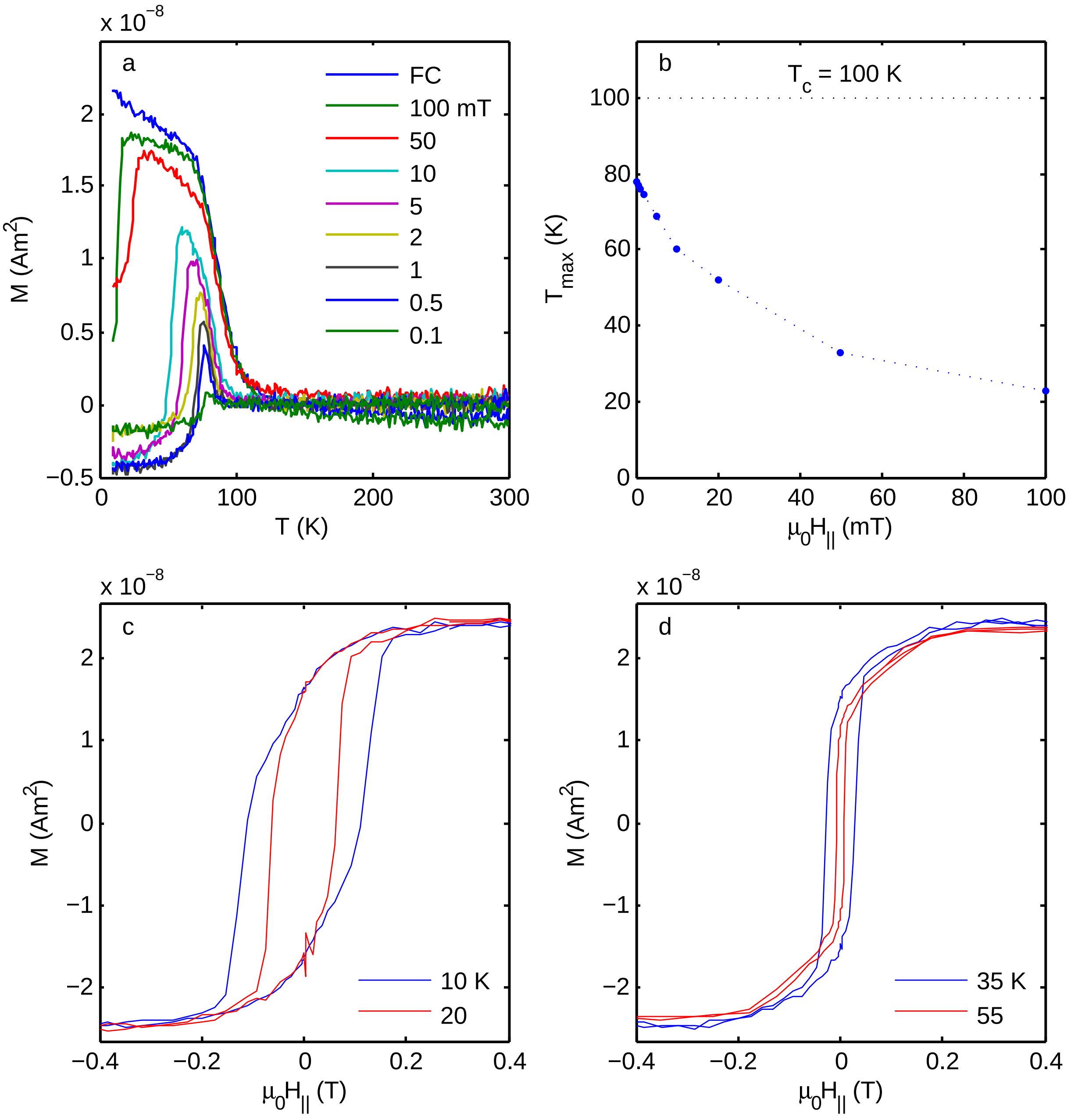

Supplementary Figure 5: Temperature dependent in-plane magnetic properties of N=12 u.c. sample. (a) Field cooled (FC) and zero-field cooled (ZFC) in-plane magnetic moment $M(T)$ measured in different applied measurement fields $\mu_0 H_{\|}$. (b) T_{max} vs. $\mu_0 H_{\|}$ showing decrease of T_{max} with field. (c,d) $M(H)$ loops at various temperatures showing the decrease in the coercive field with temperature.

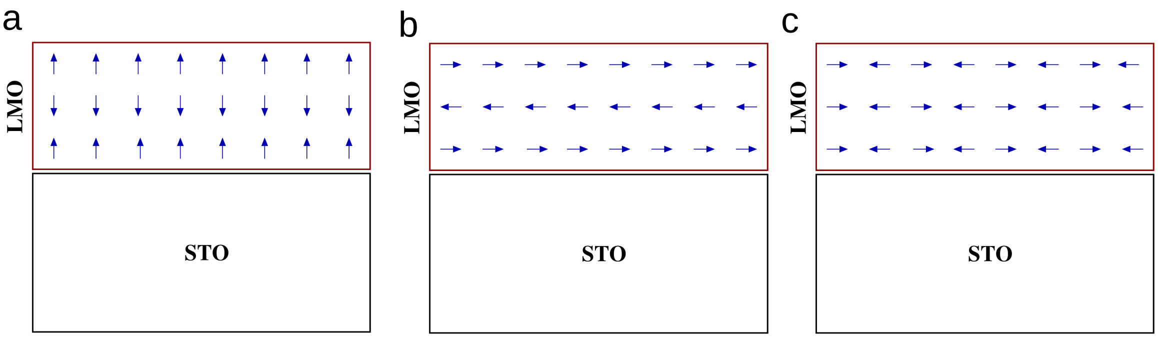

Supplementary Figure 6: **Possible A-type AFM arrangements in LMO.** The configuration in (c) is consistent with our measurements.

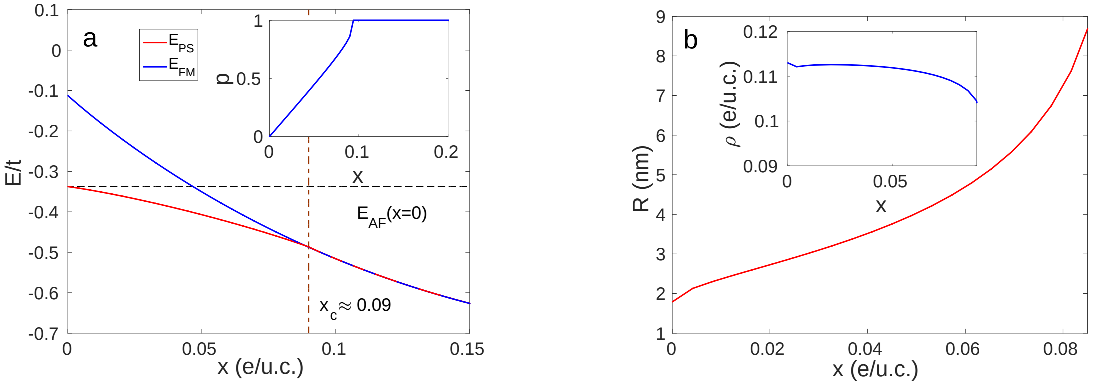

Supplementary Figure 7: **Magnetic phases in bulk LMO.** (a) Comparison of energies of the FM and phase separated states as a function of doping x in the bulk. The energy of the incompressible AFM state at $x = 0$ is shown as a reference. The FM volume fraction, p, is shown in the inset. (b) Radius of the FM puddle as a function of x. The inset shows the charge density within a FM puddle.

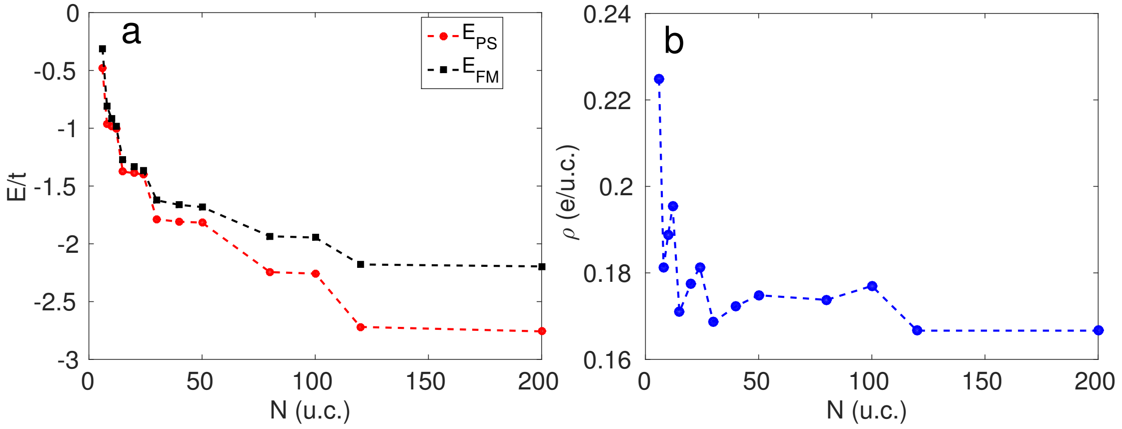

Supplementary Figure 8: **Possible magnetic states in LMO heterostructure.** (a) Comparison of energies of the FM, $E_{\rm FM}$, and phase separated, $E_{\rm PS}$, states as a function of LMO thickness showing the stability of PS state for all $N > N_c$. (b) The charge density $\rho(N)$ within a FM island in the PS state.

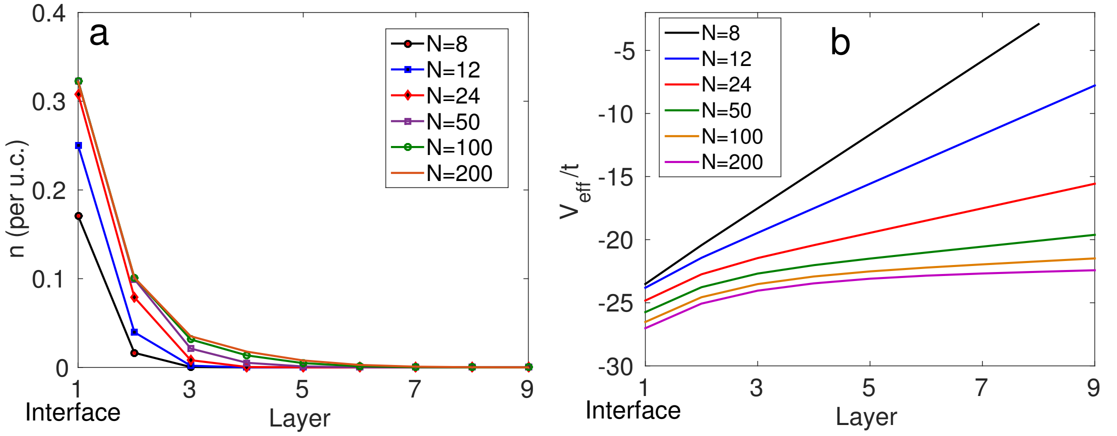

Supplementary Figure 9: **Poisson-Schrodinger calculation.** (a) Layer-resolved electron charge distribution at the LMO/STO interface. Charges only spread into a few layers (< 6) of LMO. (b) The effective potential $V_{\rm eff}(l)$ that confines the excess charges is shown for various LMO thicknesses N.

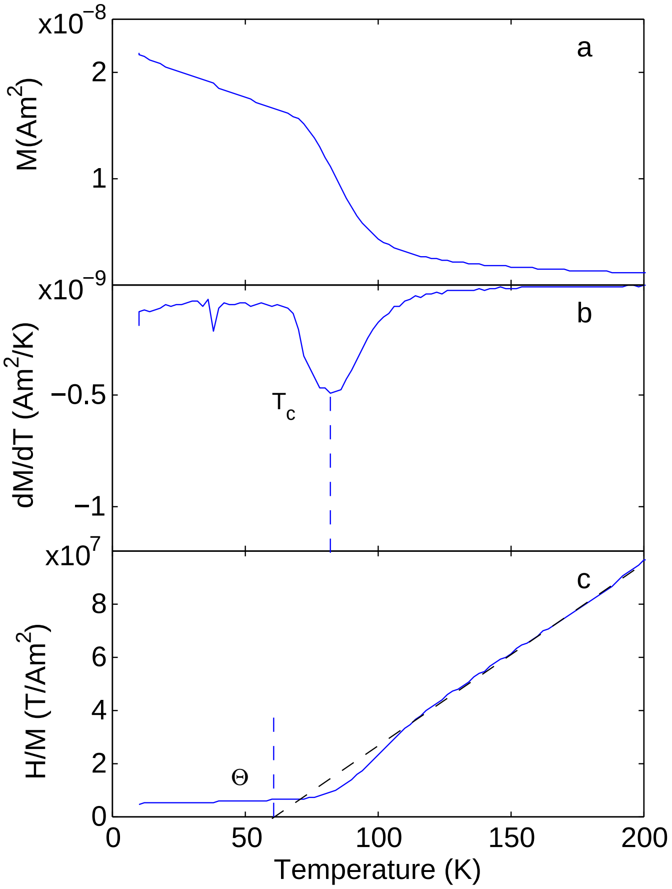

Supplementary Figure 10: **Currie-Weiss behavior in $N = 12$ u.c. sample.** (a) In-plane magnetic moment $M(T)$ measured in $\mu_0 H_{\|} = 1$ T. (b) dM/dT vs. T showing the critical temperature T_c. (c) Inverse of high field susceptibility H/M showing the Currie-Weiss scaling at high temperature (dashed line) and the extrapolated temperature Θ.

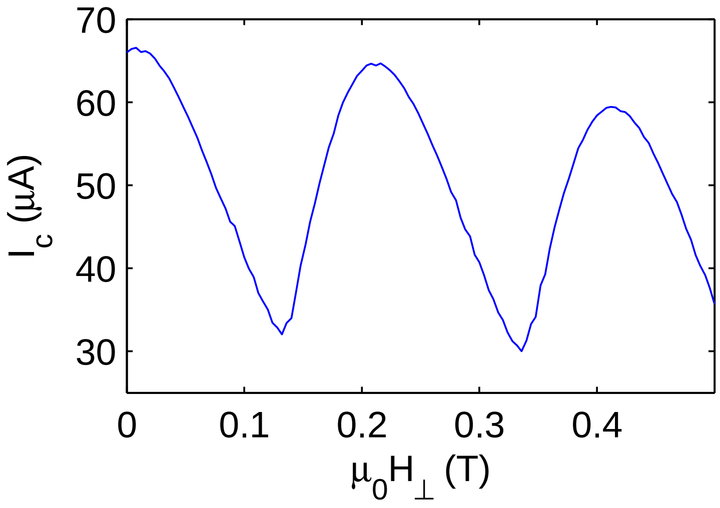

Supplementary Figure 11: **Quantum interference pattern of a Pb SOT**. Critical current $I_c(H_\perp)$ of the SOT used for measurement of the 8 u.c. sample vs. out-of-plane magnetic field at 4.2 K.

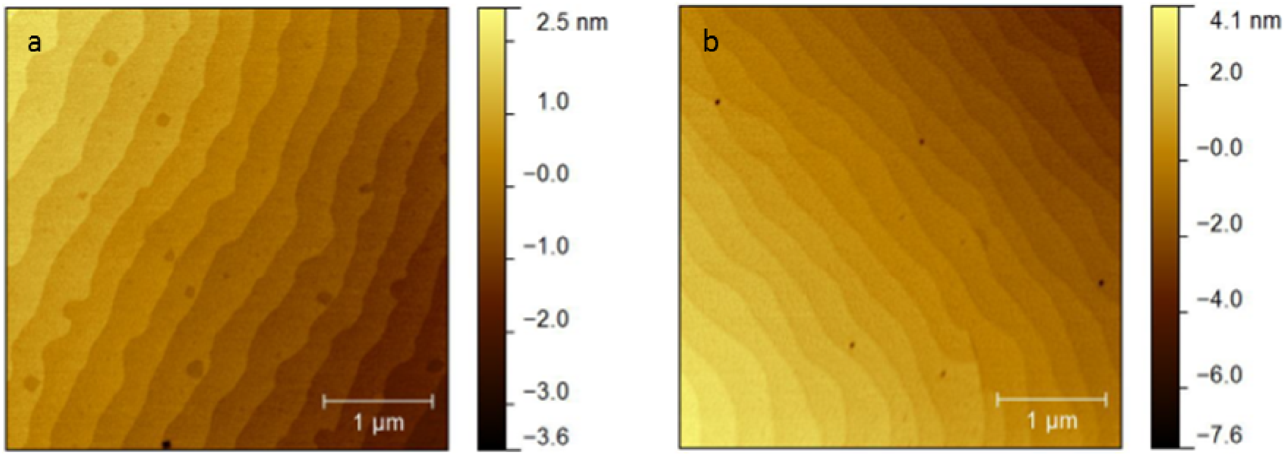

Supplementary Figure 12: **Surface topography of the LMO/STO samples.** Atomic force microscopy of the 12 u.c. (a) and 24 u.c. (b) samples showing single atomic step terraces.

Sample (u.c.)	4	5	6	8	12	24	200
SOT diameter (nm)	229	229	101	114	104	90	111
$\mu_0 H_\perp$ (mT)	10	20	28	28	65	142	65
h (nm)	$\sim$100	$\sim$100	80	105	105	137	$\sim$150

Supplementary Table 1: **SOT parameters for various samples.** Listed are the SOT diameters, the applied out-of-plane field $\mu_0 H_\perp$ at the working point, and the estimated scanning height h of the SOT above the sample surface.

Supplementary Note 1: Additional $B_z(x, y)$ and $\Delta B_z(x, y)$ images

We explored several different regions of the samples with no qualitative differences. Supplementary Figure **2** shows a large area $B_z(x, y)$ scan of 10×10 μm^2 of the $N = 12$ u.c. sample after ZFC, demonstrating the relative uniformity of the magnetic features.

Supplementary Figure **3** shows examples of the differential $\Delta B_z(x, y)$ images in various samples. All the samples with $N > N_c = 5$ show clear dipole-like features of SPM reversal events. For the $N = 200$ u.c. sample, our maximal $\mu_0 H_{\|} = 250$ mT was insufficient to reach H_c in order to study SPM reversals.

Supplementary Note 2: Global magnetization measurements

Global magnetization measurements of the samples were done using a Quantum Design magnetic properties measurement system (MPMS) vibrating sample magnetometer. Supplementary Figures **1**a,b show the magnetic hysteresis $M(H)$ loops for LMO samples of different thickness N. The 'STO' curve refers to a bare STO substrate that went through the same process, not including PLD. The finite hysteretic signal of the bare STO may either arise from an artifact such as residual magnetic field of the magnetometer's superconducting magnet[1] or from silver paint contamination of the substrate[2].

The $N = 4$ and 5 u.c. samples show a very small change in magnetization relative to the bare STO, while a substantial difference is observed upon increasing the thickness by a single u.c. to $N = 6$, as shown in Supplementary Figure **1**c. The saturation magnetic moment M_s (as well as the coercive field) increases monotonically with $N > N_c = 5$. Supplementary Figure **1**c also presents M_s per Mn atom, which shows a sharp jump at $N = 6$ and a non-monotonic behavior at larger thicknesses. The magnetization per Mn atom is always smaller than the expected $4\mu_B$ indicating that only a fraction of the Mn atoms are in the FM state.

The temperature dependence of the in-plane magnetic properties of $N = 12$ u.c. sample are shown in Supplementary Figure **5**, revealing the onset of magnetism below T_c=100K. Field cooling (FC) was done using a cooling field $\mu_0 H_{\|} = 1$ T and a measurement field of $\mu_0 H_{\|} = 0.1$ T was applied during the warm-up process. Zero field cooling (ZFC) measurements were done during warm-up in the presence of the indicated measurement field values. As shown in Supplementary Figure **5**a, ZFC curves display a maximum at T_{max} which decreases with $H_{\|}$ as summarized in Supplementary Figure **5**b. In addition, magnetic hysteresis loops (Supplementary Figures **5**c and **5**d) acquired at different temperatures show that the coercive field $\mu_0 H_c$ decreases with increasing temperature, down to 10 mT at 55 K. The behavior of T_{max} and the hysteresis loops point to a possible existence of a blocking temperature $T_B \gtrsim$80 K [3–7].

In order to evaluate the existence of AFM ordering, Supplementary Figure **10**a shows the high-field magnetic moment M vs. T in $N = 12$ u.c. sample measured in 1 T field. The critical temperature $T_c = 82$ K is estimated from peak position in the derivative of the magnetization dM/dT as shown in Supplementary Figure **10**b. The inverse of the magnetic susceptibility H/M in Supplementary Figure **10**c shows a clear Currie-Weiss behavior with extrapolated $\Theta = 61$ K. The fact that $\Theta < T_c$ is a clear indication of AFM interactions in the sample [8].

Supplementary Note 3: In-plane anisotropy

By applying $H_{\|}$ at different angles, we find a significant in-plane magnetic anisotropy of the SPM islands. For $H_{\|}$ oriented close ($\theta = 7°$) to the [100] STO direction (x-axis), the angular distribution of the SPM magnetization reversals is peaked at $\theta = 0$, as shown in Supplementary Figure **4**a and illustrated by the $\Delta B_z(x, y)$ image in Supplementary Figure **4**d. For $H_{\|}$ at 52°, most of the events are still oriented around $\theta = 0°$ (Supplementary Figures **4**b,e). However, few events appear at angles close to $\theta = 90°$. When $H_{\|}$ is at 97° (Supplementary Figures **4**c,f), the angular distribution shows a broad maximum around the y-axis ([010] STO). The in-plane magnetization thus shows fourfold anisotropy with fourfold easy axes along the LMO crystallographic directions that are locked to the underlying STO crystal structure. The observed differences in the anisotropy barrier for the two orthogonal directions is caused apparently by symmetry breaking at the cubic-to-tetragonal transition of STO at $T < 105$ K, leading to domain structure [9].

Supplementary Note 4: Theoretical model for magnetism in LMO/STO heterostructure

Phase separation in bulk LMO

We first give a simple theoretical description of the phase separation phenomena and formation of ferromagnetic (FM) islands in bulk LMO following Ref.10. As discussed in the main text, the 'A-type' antiferromagnetic (AFM) state of undoped LMO consists of FM planes that are aligned antiferromagnetically [11–14]. The AFM state can be described by the Hamiltonian

$$\mathcal{H}_0 = -J_F \sum_{i,\mu} \mathbf{S}_i \cdot \mathbf{S}_{i+\hat{\mu}} + J_{AF} \sum_i \mathbf{S}_i \cdot \mathbf{S}_{i+\hat{\nu}} - J_{\mathrm{H}} \sum_i \mathbf{S}_i \cdot \mathbf{s}_i, \tag{1}$$

where i is the position of the Mn^{3+} ions on a simple cubic lattice with spacing $a = 0.39$ nm, $\hat{\mu}$ denotes the directions in the FM planes and $\hat{\nu}$ the out-of-plane AFM direction. $\mathbf{S}_i$ and $\mathbf{s}_i$ are the core spin ($S = 3/2$) and e_g electron spin, respectively, coupled via Hund's coupling J_{H}. We work with $J_F = J_{AF} = J > 0$ and in the limit $J_{\mathrm{H}} \to \infty$. The AFM in LMO is slightly canted, leading to a small magnetic moment $\sim 0.2\ \mu_{\mathrm{B}}$ per u.c. due to Dzyaloshinskii-Moriya exchange [12, 15]. We incorporate this by assuming a background magnetic moment of $\sim 0.2\ \mu_{\mathrm{B}}$ per u.c. while estimating the saturation magnetization of the sample.

LMO in the bulk can be doped by injecting excess e_g electrons or holes chemically, e.g. by doping with Ce or Sr, respectively. Alternatively oxygen excess, e.g. induced during the growth of LMO thin film, could give rise to similar effect. The kinetic energy of the carriers in doped LMO is described by the 'double exchange' model [14]

$$\mathcal{H}_{\mathrm{kin}} = -t \sum_{\langle ij \rangle} \cos\left(\frac{\theta_i - \theta_j}{2}\right) (a_i^\dagger a_j + \mathrm{h.c.}). \tag{2}$$

Here θ_i is the polar angle of the core spin and t is the hopping amplitude of the carriers (a_i). The above term prefers the core spins to align ferromagnetically ($\theta_i = \theta_j$), and thereby tends to induce metallicity. We take $t = 0.3$ eV and $J = 0.1t$ [14] for our calculations.

The competition of FM double exchange with the AFM superexchange is believed to be at the root of the nanoscale phase separation in doped manganites [14, 16, 17]. In the PS state, the long-range Coulomb interaction between non-uniform excess charge distributions plays a crucial role in determining the typical scale of the phenomenon.

We consider the phase separation in bulk LMO doped with x electrons per Mn. In the low doping regime of interest here, we assume that the excess electrons segregate to form a periodic arrangement of spherical FM islands or puddles of radius R with a density ρ (per site) within an undoped AF background. The periodic arrangement is defined by a cubic u.c. of volume $(4\pi/3)R^3/p$, where p is the FM volume fraction that determines the average spacing $\sim R/p^{1/3}$ between FM islands. The u.c. have a neutralizing uniform positive charge density xe corresponding to the dopants and the charge neutrality condition implies $\rho = x/p$. The energy of the phase separated state can be written as, $E_{\rm PS} = E_{\rm Kin} + E_{\rm Mag} + E_{\rm Coulomb}$, where $E_{\rm Kin}$ corresponds to the kinetic energy of the electrons confined within the FM metallic island and can be easily estimated[10]; $E_{\rm Mag} = -(2J_F + J_{AF})S^2 + 2J_{AF}S^2p$ is the magnetic exchange energy of the phase separated state. We take $J_F = J_{AF} = 0.1t$, with $t = 0.3$ eV [14]. The Coulomb energy cost is entirely due to the charging energy of each spherical u.c., as there is no inter-island interaction in this approximation, and could be obtained as

$$E_{\rm Coulomb} = \frac{2\pi}{5} V x^2 \left(\frac{R}{a}\right)^2 (2 - 3p^{1/3} + p)/p \tag{3}$$

where $V = e^2/\epsilon a$ is the strength of Coulomb interaction that is estimated by using static dielectric constant $\epsilon \approx 100$ for doped manganites at low temperature and low-frequency [18]. We obtain the optimal size of the FM island by minimizing $E_{\rm PS}$ with respect to R and p. As evident in Supplementary Figure **7**a, the phase separated state has lower energy than FM for $0 \lesssim x \lesssim x_c \approx 0.1$. As shown in the inset, the FM volume fraction $p \to 1$ as $x \to x_c$ and whole system becomes a uniform FM beyond $x = x_c$. Supplementary Figure **7**b shows the radius R as function of doping; R increases with x and diverges approaching the transition to the uniform FM. The diameter $(2R)$ of the FM island is between $4 - 20$ nm. The inset of Supplementary Figure **7**b implies that the charge density ρ within the FM island varies weakly as a function of x and stays close to the critical density $x_c \approx 0.1$.

Charge reconstruction in LMO/STO heterostructures

As discussed in the main text, due to the polar nature, LMO/STO heterostructure can undergo an electronic reconstruction as in LAO/STO [19]. As a result, the heterostructure consists of an electron-doped layer within the LMO near the interface and a hole doped layer at the top surface [20]. We estimate the charge density qe (per 2D u.c.) of doped LMO layers using $q(N) = 0.5(1 - N_c/N)$ (Fig. 5e), where we take the critical thickness $N_c = 5$ in conformity with experiment. This simple form can be obtained in the intrinsic polar catastrophe scenario [20]; however, here we treat it as an empirical formula. Since our model is electron-hole symmetric, from here on, we only refer to the electron-doped layer.

Charge distribution in LMO: Schrödinger-Poisson calculation

The excess charges are confined close to the surface and interface due to electrostatics. However, they can lower their kinetic energy by delocalizing in the z-direction. We self-consistently obtain the spread N_e of the electrons from the interface along the z-direction by performing a Schrödinger-Poisson calculation, assuming a single hole-doped layer with charge $+qe$ per 2D u.c. as a boundary condition at the top surface. This gives us an estimate of the layer-resolved charge distribution $n(l)$, l being the layer index, and the effective single-particle potential $V_{\rm eff}(l)$ that confines the electrons near the interface. The electric field (in the z-direction) between layers l and $l+1$ is $\mathcal{E}(l, l+1) = \mathcal{E}_{\rm pol} + \mathcal{E}_{\rm S} + \mathcal{E}_{\rm H}$,

where $\mathcal{E}_{\rm pol} = 2\pi e/\tilde{\epsilon}a^2$ is the electric field due to alternating polar LaO^+ and $MnO_2{}^-$ sublayers, $\mathcal{E}_{\rm S} = -2\pi qe/\tilde{\epsilon}a^2$ the field due to the hole-doped layer at the surface and

$$\mathcal{E}_{\rm H}(l, l+1) = -\frac{2\pi qe}{\tilde{\epsilon}a^2} + \frac{4\pi e}{\tilde{\epsilon}a^2} \sum_{j=l+1}^{N} n(j) \quad (4)$$

is the electric field due to the Hartree potential for the charge distribution $\{n(l)\}$. Here $\tilde{\epsilon} \simeq 18$ [18] is the low temperature dielectric constant of bulk undoped LMO. The potential $V_{\rm eff}(l)$ is obtained by summing over the fields from the interface to the l-th layer. The kinetic energy is given by $\mathcal{H}_0 = \sum_{\mathbf{k},ll'} \epsilon_{ll'}(\mathbf{k}) a^\dagger_{\mathbf{k}l} a_{\mathbf{k}l'}$, where the energy dispersion $\epsilon_{ll'}(\mathbf{k})$ contains the z-direction hopping t and the 2D dispersion in the xy-plane, $\epsilon_0(\mathbf{k}) = -2t(\cos k_x a + \cos k_y a) \approx -4t + ta^2k^2$, with $\mathbf{k} = (k_x, k_y)$. We work with spinless Fermions, as appropriate for the double exchange model (see below) assuming a uniform FM phase for the doped layers. The Hamiltonian $\mathcal{H}_0 + V_{\rm eff}$ is diagonalized starting with an initial charge distribution $\{n(l)\}$ and $n(l)$ is obtained self-consistently via $n(l) = \sum_{\mathbf{k}\lambda} n_{\rm F}(\varepsilon_\lambda(\mathbf{k}))\, |\psi_{\lambda l}(\mathbf{k})|^2$, where $\varepsilon_\lambda(\mathbf{k})$ and $\psi_{\lambda l}(\mathbf{k})$ are the eigenvalues and eigenfunctions, respectively, and $n_{\rm F}$ is the Fermi function. The Fermi energy is determined by the charge neutrality constraint $\sum_{l=1}^{N} n(l) = q$. The results for $n(l)$ and $V_{\rm eff}(l)$ are shown in Supplementary Figure **9**. Since the charge density profile decays exponentially with the number of layers, to determine the number of doped layers we used a cut-off of $n = 0.005$. Below we show that the doped layers lead to a phase-separated (PS) state exhibiting superparamagnetism.

Phase separation in LMO/STO heterostructures

As in the case of bulk LMO, we estimate various contributions to the energy of the PS state as a function of the FM area fraction p_a and the radius R of the islands in the 2D case of the LMO/STO heterostructure.

Kinetic energy: We estimate the kinetic energy $E_{\rm kin}(R, p_a)$ of the electrons within the FM island subjected to the effective confining potential $V_{\rm eff}(l)$. The kinetic energy of the electrons confined within an area πR^2 in the xy plane is obtained from $\mathcal{H}_0 = \sum_{\mathbf{n},ll'} \epsilon_{ll'}(\mathbf{n}) a^\dagger_{\mathbf{n}l} a_{\mathbf{n}l'}$, where $\mathbf{n} = (n_x, n_y)$; n_x, n_y being positive integers and $\epsilon_{ll'}(\mathbf{n})$ contains z-direction hopping t and 2D particle-in-a-box energy levels $\epsilon_0(\mathbf{n}) \approx -4t + ta^2\pi(n_x^2 + n_y^2)/R^2$ for a box of linear dimension $\sqrt{\pi}R$. By diagonalizing $\mathcal{H}_0 + V_{\rm eff}$, we obtain the kinetic energy of the electrons $E_{\rm kin}(R, p_a)$ as a function of R and the FM fraction p_a.

Magnetic energy: The formation of FM islands, while reducing the kinetic energy, leads to loss of magnetic exchange energy, which essentially limits the FM area fraction p_a. As shown in Supplementary Figure **6**, there are three possible A-type AFM arrangements for the LMO/STO structure. If the spin configurations of Supplementary Figures **6**a **6**b are realized, then one expects to see a large magnetic signal from different AFM domains in the SOT scans for odd number of LMO layers for $N \leq N_c$, in contrast to our observations (Fig. 1). Also, the configuration of Supplementary Figure **6**b is highly unlikely as our SOT measurements find that the SPM islands have in-plane magnetic moment. Therefore, for our calculations, we consider the spin configuration of Supplementary Figure **6**c. In principle, the AFM configuration in LMO/STO heterostructure for $N \leq N_c$ could be different from the A-type AFM in the bulk, e.g. G-type or C-type. However, the qualitative fact that we obtain an inhomogeneous SPM state for all $N \leq 200$ will not change if we take G-type of C-type AFM states as FM tendencies will be even more suppressed.

For $N > N_c$, N_e layers get doped with electrons. If these layers host FM islands in an AFM matrix with a FM area fraction p_a, then the magnetic energy of the N_e layers is given by $E_{\rm mag}(p_a) =$

$-(3N_e - 2p_aN_e - 1)JS^2$. As in the case of bulk LMO, the competition between kinetic double exchange and magnetic superexchange gives rise to a PS state with $p_a < 1$. However, as the excess charges segregate within the FM regions, it costs a lot of Coulomb energy to form a large FM region. This essentially limits the size of the FM islands.

Coulomb energy: To obtain the Coulomb energy cost, we approximate the hole-doped layer at the surface as a uniformly charged 2D plane with surface charge density $\sigma_0 = qe/a^2$ and the electron doped layer at the interface as a square lattice of 2D disks, with radius R and surface charge density $\sigma_f = -\sigma_0/p_a$, having average spacing $(\pi/p_a)^{1/2}R$. The Coulomb energy is obtained from $E_{\rm Coulomb} = (\pi/\epsilon L^2)\int dk_z \sum_{\mathbf{k}_\parallel} |\rho(\mathbf{k})|^2/k^2$, where $\mathbf{k} = (\mathbf{k}_\parallel, k_z)$, L^2 is the area of the system, and $\rho(\mathbf{k})$ is the Fourier transform of the 3D charge density. For FM area fraction $p_a < 1$, the Coulomb energy (per 2D u.c.) contribution from the non-uniform part of the charge distribution is obtained as

$$E_{\rm Coulomb} = 4\pi V q^2 \left(\frac{R}{a}\right) \frac{1}{p_a^{3/2}} \sum_{\mathbf{g}\neq 0} \frac{J_1^2(\sqrt{p_a}g)}{g^3}, \tag{5}$$

where $\mathbf{g} = 2\sqrt{\pi}(g_1\hat{\mathbf{x}} + g_2\hat{\mathbf{y}})$, g_1, g_2 being integers, and $J_1(x)$ the Bessel function, and $V = e^2/\epsilon_{\rm PS}a$ is determined by the dielectric constant $\epsilon_{\rm PS}$ in the PS state. Since $\epsilon_{\rm PS}$ is not known, we take for our calculation $\epsilon_{\rm PS} = \epsilon \approx 100$, the value for doped LMO [18]. However, our results do not change qualitatively over a range of $\epsilon_{\rm PS}$ values.

Numerical Results

Summing over $E_{\rm mag}(p_a)$, $E_{\rm kin}(R, p_a)$, and $E_{\rm Coulomb}(R, p_a)$, we obtain the energy $E_{\rm PS}(p_a, R)$ of the PS state and minimize it to obtain the optimal diameter D and area fraction p_a of the FM islands, as shown in figures. 5f and 5g. The magnetic moment m (Fig. 5f) of the FM islands is obtained from their volume $\pi R^2 N_e a$ assuming $4\mu_{\rm B}$ per Mn atom. The total magnetic moment M of the sample (Fig. 1g) is calculated by summing the magnetic moments m of the electron- and hole-doped layers over the 5×5 mm^2 area of the sample, as well as the background contribution of $0.2\mu_{\rm B}$ per Mn for the $(N-2N_e)+2(1-p_a)N_e$ undoped AFM part of the LMO layers. Energies of the SPM and FM states are compared in Supplementary Figure **8**a. We find the SPM state to be stabilized over uniform FM, i.e., $p_a < 1$, for all thicknesses $6 \le N \le 200$, in conformity with our SOT measurements. The charge density inside each FM island varies weakly with N for $N > 6$ and stays around 0.17 (Supplementary Figure **8**b). Figure 5f shows that the size of the FM islands is on the nm scale, giving rise to the SPM behavior. The calculated moments and diameters of the FM islands are in good agreement with corresponding typical values, $D \simeq 19$ nm and $m \simeq 1.5\times 10^4$ $\mu_{\rm B}$, found experimentally (Fig. 3j). However, in reality, disorder can give rise to a distribution of these quantities, as seen in figure. 3j. The quantities D, m, and p_a show non-monotonic dependence on N, peaking at $N \simeq 12$ (Fig. 5f,g). Around this thickness, a transition from insulating SPM to the metallic FM state could be induced by increasing the carrier concentration at the interface by an external gate voltage.

Supplementary Note 5. SQUID Characteristics

The scanning SOT microscopy technique, including the Pb SOT fabrication and characterization, is described in Refs. 21, 22 and 23. Supplementary Figure **11** shows the measured quantum interference pattern $I_c(H_\perp)$ of the Pb SOT used to investigate the 8 u.c. sample, which is typical for our devices. It had an effective diameter of 114 nm (204 mT modulation period), 66 μA critical current at zero field, and white flux noise (at frequencies above a few hundred Hz) of 200 $n\Phi_0$Hz$^{-0.5}$. A different

SOT of ~ 100 nm diameter was used for each sample to study the local $B_z(x, y)$, as summarized in Supplementary Table **1**. Since the 4 and 5 u.c. samples produced a very weak signal, a larger SOT of 229 nm was used for both samples.

SOTs are sensitive only to the out-of-plane component of the magnetic field B_z and can operate in the presence of elevated in-plane and out-of-plane fields. The field sensitivity of a SOT arises from the field dependence of its $I_c(H_\perp)$ and is maximal around the regions of large $|dI_c/dH|$. Therefore, the SOTs usually have poor sensitivity at $H_\perp = 0$, as seen from Supplementary Figure **11**. Using a vector magnet, we have applied a constant $H_\perp$ to bias the SOT to a sensitive region and then imaged the local $B_z(x, y)$ at various values of $H_\parallel$ up to our highest field $\mu_0 H_\parallel = 250$ mT. The presence of $H_\perp$ did not cause any observable effect on $B_z(x, y)$ because of the in-plane magnetization of LMO with large anisotropy. The values of the applied $H_\perp$ for the various samples are listed in Supplementary Table **1** along with the estimated scanning height h of the SOT above the sample surface. For 6 to 24 u.c. samples, we have a more accurate evaluation of h, obtained from the best fit to $\Delta B_z(x, y)$, as demonstrated in Fig. 2d and described in method.

References

[1] Quantum Design 2009 Application Note 1070-207: Using PPMS Superconducting Magnets at Low Field.

[2] Golmar, F., Mudarra Navarro, A. M., Rodríguez Torres, C. E., Sánchez, F. H., Saccone, F. D., dos Santos Claro, P. C., Benítez, G. A., Schilardi, P. L. Extrinsic origin of ferromagnetism in single crystalline $LaAlO_3$ substrates and oxide films. *Applied Physics Letters*, **92**, 262503 (2008).

[3] Wernsdorfer, W. (2001) Classical and Quantum Magnetization Reversal Studied in Nanometer-Sized Particles and Clusters, in Advances in Chemical Physics, Volume 118 (eds I. Prigogine and S. A. Rice), John Wiley & Sons, Inc., Hoboken, NJ, USA.

[4] Shinde, S. R., Ogale, S.B., Higgins, J.S. Zheng, H., Millis, A. J., Kulkarni,V. N., Ramesh, R.,Greene, R. L., Venkatesan, T. Co-occurrence of Superparamagnetism and Anomalous Hall Effect in Highly Reduced Cobalt-Doped Rutile TiO_2 Films. *Physical Review Letters*, **92**, 166601 (2004)

[5] Chen, Q., Rondinone, A.J., Chakoumakos, B.C., Zhang, Z.J. Synthesis of superparamagnetic $MgFe_2O_4$ nanoparticles by coprecipitation. *Journal of Magnetism and Magnetic Materials*, **194**, 1-7 (1999)

[6] Zhang,Y.D., Budnick, J.I., Hines, W. A., Chien, C.L., Xiao, J.Q. Effect of magnetic field on the superparamagnetic relaxation in granular Co-Ag samples. *Applied Physics Letters*, **72** 2053-2055 (1998)

[7] Bitoh, T., Ohba, K., Takamatsu, M., Shirane, T., Chikazawa, S. Field-cooled and zero-field-cooled magnetization of superparamagnetic fine particles in $Cu_{97}Co_3$ alloy: comparison with spin-glass $Au_{96}Fe_4$ alloy. *Journal of the Physical Society of Japan*, **64**, 1305-1310 (1995)

[8] Prado, F., Sánchez, R. D., Caneiro, A., Causa, M. T. & Tovar, M. Discontinuous Evolution of the Highly Distorted Orthorhombic Structure and the Magnetic Order in $LaMnO_{3\pm\delta}$ Perovskite. *J. Solid State Chem.* **146**, 418–427 (1999).

[9] Honig, M., Sulpizio, J. A., Drori, J., Joshua, A., Zeldov, E., Ilani, S. Local electrostatic imaging of striped domain order in $LaAlO_3/SrTiO_3$. *Nature Materials* **12**, 1112-1118 (2013).

[10] Gonzalez, I., Castro, J. & Baldomir, D. On the absence of conduction electrons in the antiferromagnetic part of the phase-separated states in magnetic semiconductors. *Phys. Lett. A* **298**, 185-192 (2002).

[11] Moreo, A., Yunoki, S. & Dagotto, E. The Phase Separation Scenario for Manganese Oxides. *Science* **283**, 2034–2040 (1999).

[12] Salamon, M. B. & Jaime, M. The physics of manganites: Structure and transport. *Rev. Mod. Phys.* **73**, 583–628 (2001).

[13] Coey, J. M. D., Viret, M. & von Molnár, S. Mixed-valence manganites. *Adv. Phys.* **48**, 167–293 (1999).

[14] Dagotto, E. *Nanoscale Phase Separation and Colossal Magnetoresistance* (Springer Berlin Heidelberg, 2003). doi:10.1007/978-3-662-05244-0

[15] Skumryev, V., Ott, F., Coey, J. M. D., Anane, A., Renard, J. P. & Revcolevschi, A. Weak ferromagnetism in $LaMnO_3$. *Eur. J. Phys. J. B* **11**, 401-406 (1999).

[16] Nagaev, E. L. New type of self-localization state of carriers in an antiferromagnetic semiconductor. *Pis'ma Zh. Eksp. Teor. Fiz.* **55**, 646 (1992).

[17] Kagan, M. Y. & Kugel, K. I. Inhomogeneous charge distributions and phase separation in manganites. *Physics-Uspekhi* **44**, 553 (2001).

[18] Cohn, J. L., Peterca, M. & Neumeier, J.J., Low-temperature permittivity of insulating perovskite manganites. *Phys. Rev. B* **70**, 214433-(1-6) (2004).

[19] Hwang, H. Y. *et al.* Emergent phenomena at oxide interfaces. *Nat. Mater.* **11**, 103–113 (2012).

[20] Wang, X. R. et al. Imaging and control of ferromagnetism in a polar antiferromagnet. *Science* **349**, 716–719 (2015)

[21] Finkler, A. *et al.* Self-aligned nanoscale SQUID on a tip. *Nano Lett.* **10**, 1046–1049 (2010).

[22] Finkler, A., Vasyukov, D., Segev, Y., Neeman, L., Lachman, E. O., Rappaport, M. L., Myasoedov, Y., Zeldov, E., Huber, M. E. Scanning superconducting quantum interference device on a tip for magnetic imaging of nanoscale phenomena. *Review of Scientific Instruments*, **83**, 073702 (2012)

[23] Vasyukov, D. *et al.* A scanning superconducting quantum interference device with single electron spin sensitivity. *Nat. Nanotechnol.* **8**, 639–44 (2013).
